# Progress with viral vectored malaria vaccines: A multi-stage approach involving “unnatural immunity”

**DOI:** 10.1016/j.vaccine.2015.09.094

**Published:** 2015-12-22

**Authors:** Katie J. Ewer, Kailan Sierra-Davidson, Ahmed M. Salman, Joseph J. Illingworth, Simon J. Draper, Sumi Biswas, Adrian V.S. Hill

**Affiliations:** a The Jenner Institute, University of Oxford, Oxford OX3 7DQ, UK; b Vaccine Research Center (VRC), National Institute of Allergy and Infectious Diseases, National Institutes of Health, Bethesda, MD 20852, USA

**Keywords:** Viral vectors, Malaria, Immunity, Clinical trials

## Abstract

Viral vectors used in heterologous prime-boost regimens are one of very few vaccination approaches that have yielded significant protection against controlled human malaria infections. Recently, protection induced by chimpanzee adenovirus priming and modified vaccinia Ankara boosting using the ME-TRAP insert has been correlated with the induction of potent CD8^+^ T cell responses. This regimen has progressed to field studies where efficacy against infection has now been reported. The same vectors have been used pre-clinically to identify preferred protective antigens for use in vaccines against the pre-erythrocytic, blood-stage and mosquito stages of malaria and this work is reviewed here for the first time. Such antigen screening has led to the prioritization of the *Pf*RH5 blood-stage antigen, which showed efficacy against heterologous strain challenge in non-human primates, and vectors encoding this antigen are in clinical trials. This, along with the high transmission-blocking activity of some sexual-stage antigens, illustrates well the capacity of such vectors to induce high titre protective antibodies in addition to potent T cell responses. All of the protective responses induced by these vectors exceed the levels of the same immune responses induced by natural exposure supporting the view that, for subunit vaccines to achieve even partial efficacy in humans, “unnatural immunity” comprising immune responses of very high magnitude will need to be induced.

The progress of viral vectored vaccines for malaria through the clinical development pathway has accelerated considerably over the past 5 years, in particular for chimpanzee adenovirus serotype 63 (ChAd63) and modified vaccinia Ankara (MVA) vectors encoding ME-TRAP, a pre-erythrocytic vaccine construct. ME-TRAP is a multi-epitope (ME) string of known CD4^+^ and CD8^+^ epitopes from various pre-erythrocytic antigens, fused to thrombospondin-related adhesion protein (TRAP). Clinical trials have demonstrated the ability of these vectors to induce high frequencies of CD8^+^ T cells associated with the induction of useful levels of efficacy in the controlled human malaria infection (CHMI) model [1] and a field trial in a malaria-endemic population [2]. Recent data from as yet unpublished Phase I studies in children and infants in malaria-endemic regions have shown favourable safety profiles in this target population for a malaria vaccine. In this review, we describe the current progress of pre-clinical work at the Jenner Institute in Oxford on the discovery of new antigens and vaccine constructs for the pre-erythrocytic, erythrocytic and transmission stages of the parasite life cycle, for which viral vector technology is being used, as well looking ahead at the progress of some leading malaria vectored vaccine candidates.

## 1 Identifying novel protective malaria vaccine components: pre-erythrocytic antigens

The availability of a full genome sequence of *Plasmodium falciparum* and the subsequent transcriptomic and proteomic [3], [4] analysis of rodent parasites [5] has opened new avenues for research. Several genes expressed at the sporozoite and liver-stage of *P. falciparum* have been identified that are potential targets for a pre-erythrocytic subunit vaccine. The available genomic data in combination with improved mass spectrometry analysis has facilitated the best proteome coverage to date for a pre-erythrocytic stage of the human malaria parasite, in total 1991 *P. falciparum* sporozoite proteins [6]. Sporozoite protein studies have helped substantially in identifying many new potential candidates for a pre-erythrocytic vaccine to block infection before the development of clinical symptoms. However, only a minority of these have been assessed for efficacy to date, partly because there is no very efficient way to culture pre-erythrocytic stage parasites *in vitro* [7]. In addition, because *P. falciparum* cannot readily infect small animals, screening *P. falciparum* target antigens pre-clinically is challenging without the use of humanized DRAG or knock-out liver-chimeric FRG strains of mice [8], [9].

Rodent malaria parasites are therefore generally used as models to identify vaccine targets for protective immune responses against human malaria. Although a high level of orthology and homology exists between the genes of *Plasmodium* species that infect rodents and humans [10], [11], critical differences often exist in the sequence and structure between the encoded proteins. In addition, many *P. falciparum* malaria parasite genes are absent from rodent parasite genomes, making pre-clinical efficacy studies unachievable in murine models. Generation of transgenic rodent malaria parasites expressing *P. falciparum* genes can help to circumvent problems arising from structural differences that exist between functional *P. falciparum* and rodent malaria parasite orthologs. In addition, this approach broadens the options for analyzing *P. falciparum*-specific proteins, *in vivo* [12]. There is increasing evidence that antigens other than the current two leading human malaria vaccine antigens, *Pf*CSP and *Pf*TRAP, may contribute to a protective immune response [4], [5], [13], [14] and it is likely that multiple antigens will be needed to reach very high levels of efficacy.

We have recently screened a relatively large list of pre-erythrocytic *P. falciparum* vaccine candidate antigens for protective efficacy [15]. These were identified from the literature, as well as through database mining and bioinformatic analysis aiming to identify novel *P. falciparum* vaccine candidate antigens. These have been incorporated into the ChAd63 and MVA viral vectors and immunogenicity assessed in murine models. Thirteen *P. falciparum* candidate antigens were initially chosen: LSA1, LSA3, CelTOS, UIS3, LSAP1, LSAP2, ETRAMP5, Falstatin, CSP, TRAP, HT, RP-L3 and SPECT-1. Each antigen had been shown to be either well expressed during the liver-stage of infection; a target of cell-mediated immunity in naturally exposed individuals or in those immunized with irradiated sporozoites; or a homolog had been shown to be protective in murine or non-human primate (NHP) studies. A novel challenge model was used to assess the protective efficacy of these new *P. falciparum* pre-erythrocytic vaccine candidates in mice using transgenic *Plasmodium berghei* parasites expressing *P. falciparum* genes of interest, thus enabling efficacy assessments *in vivo*. These transgenic parasites have been generated by introducing the genes encoding the antigens as an ‘additional copy’ into a neutral locus of the *P. berghei* genome, either the *Pb230p* locus on chromosome 3 or the *Pbs1* locus on chromosome 12. Mice immunized with the different *P. falciparum* vaccine candidates were challenged by intravenous injection of the transgenic *P. berghei* sporozoites expressing the cognate *P. falciparum* antigen, in order to determine protective efficacy conferred by the different vaccines after immunization.

All antigens were rank ordered in comparison to the two leading malaria candidates *Pf*CSP and *Pf*TRAP using the same challenge model. Three antigens, *Pf*LSA1, *Pf*LSAP2 and *Pf*SPECT-1, provided better protective efficacy compared to *Pf*CSP and *Pf*TRAP in inbred BALB/c mice. [15] Since different strains of mice are not equally susceptible to malaria infection [16], and to minimize the risk of using an inbred murine model as an indicator for the protective efficacy in humans, CD-1 outbred mice with diverse MHC repertoires were also used, and again *Pf*LSA1, *Pf*LSAP2 and *Pf*SPECT-1 showed better protective efficacy compared to *Pf*CSP and *Pf*TRAP. Vaccination with *Pf*UIS3, *Pf*Falstatin, *Pf*LSA3 and *Pf*ETRAMP-5 in BALB/c mice provided some degree of protection, manifest largely as a delay in the time to parasitaemia, consistent with previous work with using murine *Plasmodium* challenges [17], [18]. Surprisingly, no protection was observed after vaccination with *Pf*CelTOS despite previous reports of cross-species protection in murine models [13], [19].

We have also generated transgenic parasites expressing two *P. falciparum* antigens from the sporozoite- and liver-stages of the life cycle, based on results from the initial efficacy screening using individual antigens. Specifically, two ‘double transgenic’ parasites have been constructed expressing different combinations of two candidate antigens that showed the greatest protective efficacy in challenge experiments using the single gene transgenic *P. berghei* parasites. The first expressing the most promising two novel candidates, *Pf*LSA1 and *Pf*LSAP2, and the second expressing the two current leading pre-erythrocytic antigens, *Pf*CSP and *Pf*TRAP for comparison. It has thus been possible for the first time to assess how best to combine multi-antigen vaccines and vaccination combinations using an *in vivo* model, and to generate better protection than with a single-antigen immunization.

Of course the use of transgenic rodent parasites has limitations. A murine model with a limited repertoire of MHC-restricted epitopes that may not be representative of immunogenicity observed in human populations. By assessing efficacy and immunogenicity in outbred mice strains, we aim to reflect human immunity more accurately. Interestingly, the efficacy of the two most promising antigens, *Pf*LSA1 and *Pf*LSAP2, in CD-1 mice was comparable to that in inbred mice. It is also possible that some antigens identified as poorly immunogenic in these studies may in fact be potently immunogenic in humans. However the high cost of producing vaccines requires some means of prioritizing antigens for Phase I studies is required. Finally, the use of transgenic parasites will inevitably affect the timing and magnitude of the expression of the transgenic antigen as this will vary from expression in the native parasite strain. Yet for antigens with no *P. berghei* ortholog, this approach remains the only strategy to determine potential efficacy and is therefore a useful tool in pre-clinical vaccine development.

## 2 Identifying novel protective malaria vaccine components: blood-stage antigens

Until recently, the *P. falciparum* blood-stage antigens that have received the most attention include merozoite surface protein 1 (MSP1) [20], [21], apical membrane antigen-1 (AMA-1) [22], [23] and MSP3 [24]. These proteins were prioritized in part due to their immunogenicity either during active experimental infection [25], [26], [27], [28] or immunization with non-viable purified merozoites [29], [30], [31]. However, these vaccines have yielded disappointing (or at least uncertain) results in clinical trials, likely because naturally-acquired immunity to malaria owes its protective efficacy to strong responses against a broad range of antigens [32] and due to the polymorphic nature of these antigens. Such breadth is difficult to recapitulate with a subunit vaccine. An alternative approach involves defining the parasite antigens that are most susceptible to immune attack, regardless of their immunogenicity during natural infection. This approach was pioneered by Rappuoli and colleagues, who produced a library of more than 300 recombinant proteins from *Neisseria meningitides* serogroup B, with which they immunized rabbits to generate a large panel of antibody specificities [33]. The activity of the antibody specificities in an *in vitro* complement-mediated bacterial killing assay was used to define the most susceptible protein antigens for inclusion in a multi-component vaccine. The antigens identified in this screen are now components in the multivalent vaccine Bexsero which was licensed by the European Medicines Agency in 2013 [34]. This approach faces a number of challenges when applied to *Plasmodium* spp., primarily the difficulty of expressing and purifying recombinant protein, but also the need for much higher antibody titres to achieve efficacy against malaria compared to meningitis. For example, an attempt to bacterially express 1000 proteins from *P. falciparum* resulted in the expression of just 63 soluble proteins [35], while a separate attempt to bacterially express 95 *P. falciparum* proteins yielded just 5 soluble recombinants [36]. Recombinant viral vectors can be used to circumvent this protein production hurdle and simplify the task of obtaining high-titre, antigen-specific antibodies. Methods for the construction of recombinant viral genomes, transfection of genomes into complementary cell lines, growth of virus and subsequent purification are now well established and the materials are commercially available. Advances in production methods now mean that adenoviruses expressing transgenes whose products are deleterious to viral growth can also be produced [37]. Viral vectors therefore offer a useful ‘one-size-fits-all’ solution to the difficulties of expressing recombinant proteins and have been shown to induce functional antibodies with growth inhibitory activity [38]. Recent work undertaken at the Jenner Institute has generated viral vector pre-clinical vaccine candidates encoding 14 different proteins found on the surface of *P. falciparum* merozoites, including full-length constructs of the reticulocyte-binding protein homolog 5 (*Pf*RH5) [39], the *Pf*RH5-interacting protein (*Pf*Ripr) [40] and rhoptry-associated protein 1 (*Pf*RAP1) (unpublished data). In all cases these vaccines have elicited antibodies capable of recognizing native schizont protein in an immunofluorescence assay. *Pf*RH5 is of particular interest because previous reports that used recombinant protein fragments based on *Pf*RH5 as the immunogen did not have any significant neutralizing activity [41], [42]. By contrast, viral vectors elicited potent neutralizing polyclonal antibodies [39], and have since been used to raise the most potent neutralizing monoclonal antibodies (mAbs) yet described (as measured by the assay of GIA [40] and induced significant efficacy in a NHP model of blood-stage *P. falciparum* infection [43]. The ChAd63 and MVA vectors encoding *Pf*RH5 have since entered a Phase Ia clinical trial in Oxford (Clinicaltrials.gov NCT02181088).

While viral vectors offer considerable advantages over protein-in-adjuvant as a method for raising antigen-specific antibodies, it is important to be mindful of some caveats. These arise largely from the fact that viral vectors cause protein expression to occur *in situ* in the immunized organism, meaning that it is impossible to fully characterize the immunogen that is produced. *In silico* approaches and *in vitro* infection assays can to an extent predict protein folding, successful trafficking through the secretory pathway, polypeptide cleavage and post-translational modifications such as glycosylation, but the actual course of events in the immunized mammalian cell cannot easily be monitored in real time. On the other hand protein-in-adjuvant vaccines face similar difficulties, particularly once formulated in adjuvant. All in all, the relatively straightforward production and reliable immunogenicity of viral vectors makes them an excellent tool for the generation of antibody specificities and the definition of parasite antigens that are susceptible to antibody attack.

## 3 Identifying novel protective malaria vaccine components: transmission-blocking antigens

In a relatively new programme of work at the Jenner Institute, we have expressed leading transmission-blocking vaccine candidate antigens from ChAd63 and MVA viral vectors. Initial immunization studies with adenovirus (ChAd63 or human serotype 5 adenovirus, AdHu5) delivery vectors followed by MVA boosting, elicited anti-*Pf*s25 antibodies capable of blocking transmission in membrane-feeding assays [44]. Subsequently, we used this heterologous prime-boost vaccination regimen to directly compare the abilities of five leading sexual-stage antigens (*Pf*s25, *Pf*s230C, *Pf*HAP2, *Pf*s48/45, and AgAPN1) to induce transmission-blocking antibodies. In this screen *Pf*s25, a 25-kDa surface antigen of zygotes and ookinetes was the most potent immunogen followed by a fragment of *Pf*s230 [45]. Anti-*Pf*s25 antibodies are functional in the *ex vivo* standard membrane feeding assay (SMFA) and completely block the development of *P. falciparum* in the mosquito. The antibodies also block the development of field isolates (from gametocyte donors in Burkina Faso) of *P. falciparum*. Most recently, we have fused *Pf*s25 to IMX313, a new heptamerization technology, leading to the expression of a heptamer from viral-vectors (ChAd63 and MVA). Immunization with viral vectors expressing *Pf*s25-IMX313 shows about 10-fold greater immunogenicity and significantly better transmission-blocking efficacy in membrane feeding assays than *Pf*s25 alone [46]. In other studies elsewhere, immunization with the AdHu5 vector expressing *Pv*s25 elicited antibodies which significantly reduced the average oocyst numbers per mosquito when tested against gametocytes from *Plasmodium vivax* infected volunteers [47]; whilst antisera from mice vaccinated with baculovirus vectors (baculovirus dual-expression system) expressing both *Pv*s25 and *Pv*sCSP also elicited antibodies that gave significant transmission-blocking activity [48].

In terms of clinical trials, in 1996 volunteers were primed with a highly attenuated vaccinia viral vector expressing seven genes from *P. falciparum* (denoted NYVAC-*Pf*7), including *Pf*s25, followed by a boost of *Pf*s25 recombinant protein. The volunteers developed low level anti-*Pfs*25 antibodies which exhibited incomplete transmission-blocking activity [49]. A Phase Ia human clinical trial will be initiated in Oxford in 2015 to test viral-vectored vaccines expressing *Pf*s25-IMX313 as part of an EU funded project (MultiMalVax, see www.multimalvax.eu).

## 4 Clinical evaluation of viral vectored vaccines for malaria

Induction of CD8^+^ T cells will be a crucial component of vaccine platforms that aim to induce sterile protection against the malarial liver-stage [50]. Pre-clinical studies in mice and NHPs demonstrate that subunit platforms based on highly potent adenoviruses and MVA regimens elicit CD8^+^ T cell responses of high magnitude. This will be critical for effective clearance of all parasitized hepatocytes in order to prevent blood-stage parasitemia and associated morbidity, and mortality. Recent Phase I/II studies have translated pre-clinical immunogenicity findings with these viral vectors in order to understand vaccine-induced efficacy against malaria infection in humans, as summarized in Table 1.

### 4.1 AdHu5 CSP/AMA-1

Research at the Naval Medical Research Center in the USA led to the development of a multi-stage vaccine that combined two AdHu5 vectors containing CSP and AMA-1 (NMRC-M3V-Ad-PfCA). A Phase I/IIa study demonstrated that the vaccine was safe and well tolerated, but did not induced sterile protection in any volunteers [51], [52]. Two out of eleven vaccinees showed significant delay to patency. Notably, AdHu5 immunization induced strong CD8^+^ T cell responses against both CSP and AMA-1, largely composed of monofunctional IFNγ producers [52]. This was the first demonstration of a malaria vaccine inducing predominantly CD8^+^ T cells, and not CD4^+^ T cells, which are less directly related to protection.

### 4.2 DNA – AdHu5 CSP AMA-1

Heterologous prime-boost approaches have been shown to increase durability of CD8^+^ T cell responses and improve protection in animal models over single vaccination models [53], [54], [55]. This strategy circumvents the problem of vaccine-induced antibodies against the vector dampening boosting of immune responses upon repeated homologous vaccinations. Based on poor efficacy results with AdHu5 alone [56], the authors investigated the effect of a DNA prime prior to AdHu5 administration [57]. Vaccination with DNA AdHu5 encoding CSP and AMA-1 was safe, immunogenic and induced sterile protection in 4/15 volunteers (27% efficacy). ELISPOT responses to AMA-1 were significantly associated with protection (*p* = 0.019). Although encouraging, this level of efficacy did not differ significantly from the control group as assessed by the standard Kaplan-Meier, log rank test, analysis. AMA-1-specific IFNγ^+^ CD8^+^, but not CD4^+^, T cells were associated with the observed protection (*p* = 0.0492). Three of four sterilely protected showed higher effector to central memory CD8^+^ T cells to AMA-1 compared to non-protected volunteers (Ad5 or DNA/Ad5). Class I epitopes restricted by A*03 or B*58 within AMA-1 appeared important for protection in three of four volunteers [58]. The role of CSP in protection induced by DNA/Ad5 immunization is unknown, but changing the vector or adding additional malaria antigens may improve efficacy. Of note, administration of DNA plasmids alone expressing pre-erythrocytic-stage epitopes and administered *via* electroporation (EP1300) were assessed in a Phase I clinical trial in 2012 [59].

### 4.3 ChAd63 MVA ME-TRAP

Pre-existing neutralizing antibodies (nAbs) against human adenoviruses may interfere with protection induced by AdHu5 alone or DNA prime – AdHu5 boost vaccination. [51], [57].

Simian-derived vectors such as ChAd63 should circumvent most naturally-acquired anti-vector immunity that could diminish adenovirus-induced T cell responses [60]. The vaccine insert ME-TRAP elicits protective CD8^+^ T cell responses in mice [54], [61] and showed promising immunogenicity in NHPs [62]. Based on encouraging safety data in humans [63], a Phase I/IIa CHMI trial assessed the efficacy of ChAd63 ME-TRAP alone or followed by a boost with MVA ME-TRAP in malaria-naïve adults [1].

The clinical findings were very encouraging. Prime-boost vaccination with ChAd63-MVA induced T cell responses that were 5–10 fold higher than earlier subunit regimens with the same antigenic insert as measured by *ex vivo* IFNγ ELISPOT [64], [65]. Moreover, cellular immunity was of considerable breadth, as all volunteers recognized over half of the peptide pools spanning TRAP. The quality of T cell responses also dramatically shifted from earlier platforms. Unlike first- and second-generation viral vectors, such as fowlpox (FP9)-MVA, which predominantly induced CD4^+^ T cells [64], [65], the ChAd63-MVA combination regimen induced a high proportion of cytokine-producing CD8^+^ T cells. While no protection was induced with ChAd63 alone, the MVA boost clearly improved protective efficacy: 3/14 volunteers were sterilely protected and 5/14 showed a two-day delay in time to patent parasitemia, the latter representing a 95% reduction in liver parasite burden. Sensitive qPCR analysis revealed reduction of parasite density emerging from the liver over second and third parasite replication cycles, which negatively correlated with time to parasitemia. This provides further evidence of biological effect of the vaccine on malaria infection. Overall, ChAd63-MVA provided a total efficacy (delay plus sterile protection) of 58% (8/14), marking the first study to show statistically significant high-level, heterologous or homologous protection induced by a prime-boost regimen.

Duration of protection is as yet unclear as CHMI was performed two weeks following the final vaccination to assess short-term efficacy. Encouragingly, ELISPOT responses were detected up to 200 days post-adenovirus administration. Three sterilely protected volunteers were re-challenged eight months after their last immunization: one was sterilely protected again and two showed significant delay to patency, encouraging larger studies to further assess durability of protective immunity.

Analysis of immune responses revealed that CD8^+^ T cells secreting IFNγ, but not IL-2 or TNFα, at time of challenge correlated strongly and significantly with protection. This is line with previous studies assessing a similar construct in mice [54] and an observation in the DNA-AdHu5 trial where there was an association between frequency of the same CD8^+^ T cell population and sterile protection [57]. Notably, antibodies targeting TRAP did not appear to play a role. Pre-clinical studies in mice and NHPs have demonstrated that cellular immunity is critical for sterile protection against malaria liver-stage infection and should be sufficient in the absence of antibodies [66], [67], [68]. It is hypothesized that IFNγ secretion by CD8^+^ T cells and other innate immunity contributors such as NK and γδ T cells mediate killing of infected hepatocytes [69], [70]. However, the quality and requirements of protective CD8^+^ T cells appear to vary greatly by immunization regimen and species [69], [71]. In-depth characterization of CD8^+^ T cells induced by viral vectors in humans will be necessary to elucidate further a mechanism of protection.

ChAd63-MVA vaccination induced greater immunogenicity and efficacy compared to DNA or FP9 priming with the same antigenic insert [64], [65]. Only 9/38 volunteers showed any efficacy with either of these earlier regimens, and in the overwhelming majority of subjects, this manifested as delay to patency. This study also demonstrated a substantial improvement in antigen-specific CD8^+^ T cell frequency over DNA-AdHu5 CSP-AMA-1 vaccination [57]. The differential results of immunogenicity and efficacy may be caused by a number of reasons. First, pre-existing nAb titres against the ChAd63 vector were low. Furthermore, they did not correlate with induced T cell responses, reducing earlier concerns of anti-vector immunity in human adenovirus vaccination. Second, simian adenoviruses induce predominantly CD8^+^ T cell responses compared to FP9 and DNA priming [64], [65]: CD8^+^ T cells have been shown to directly kill infected hepatocytes *in vitro* [72]. Third, differential innate immunity elicited by viral vectors may play a critical role in shaping vaccine-induced T cells [73], [74]. Finally, the T cell responses induced by any adenovirus boosted by MVA are of substantially greater magnitude in humans than DNA- adenovirus prime-boost regimens.

Promising strategies tested in malaria-naïve individuals may fail to generate high-level immunogenicity and efficacy when deployed in endemic regions [64], [65], [75], [76]. The role of immunosuppression caused by recurrent parasitemia or interference of naturally acquired T cells and/or antibodies in vector-induced immunity is unknown. Accordingly, a Phase IIb field study was designed to assess the protective efficacy of this regimen in adults with previous malaria exposure [2]. 120 Kenyan male volunteers were administered either ChAd63-MVA ME-TRAP or the rabies vaccine and monitored for eight weeks for malaria infection. All volunteers were given anti-malarials after vaccination and prior to the PCR monitoring period in order to clear any residual parasites [77].

Immunogenicity was broadly comparable to that seen in UK vaccinees, if a little lower in magnitude. ChAd63 priming induced T cell responses approximately 5-fold greater than those induced by DNA or FP9 priming in endemic regions [75], [76]. Immune responses were biased towards IFNγ^+^ CD8^+^ T cells and detected up to six months post-vaccination, albeit by this time point they had fallen to a quarter of the peak levels. Similarities in the quantity and quality of T cell responses between exposed adults *vs.* malaria-naïve suggest that vaccination did not boost naturally-acquired immunity [78]. Interestingly, T cell responses were biased to a single TRAP peptide pool. Whether this reflects an enrichment of certain HLA alleles in the region or a mechanism of protection is unclear.

Protective efficacy was assessed by time to infection as measured by a sensitive PCR assay. In the year that the efficacy was assessed, an unexpected spike in rainfall curtailed transmission rates and decreased the overall number of infections, making it difficult to assess efficacy beyond the initial weeks of monitoring. However, Cox-regression analysis, used to analyze the primary endpoint of the trial, found that the vaccine reduced the risk of infection by 67% (95% CI 33–88%), *p* = 0.002 during the 8 weeks of monitoring. Furthermore, risk of high parasitemia (>10 parasites/mL, a secondary endpoint) was reduced by 82% (95% CI 46–94%), *p* = 0.002. The overwhelming majority of breakthrough infections were acquired during the PCR monitoring period, and were not residual or recrudescent. SNP genotyping analysis demonstrated that only 1/19 samples pre *vs.* post treatment were identical. Interestingly, once again a T cell correlate of vaccine efficacy was identified with this approach, in this case the *ex vivo* IFNγ ELISpot response to the immunodominant pool of TRAP peptides.

Efficacy in this study was much higher than an earlier Phase IIa CHMI study. There are a number of possible explanations. Firstly, there is a lower intensity of challenge inoculum in the field. CHMI requires the bites of five heavily infected mosquitoes to evaluate efficacy compared to probably a single bite in the field. In addition, transmission was unexpectedly low for the study area, decreasing the parasite liver burden and the threshold for sterile immunity. Secondly, the atovaquone component of treatment prior to the PCR monitoring period has a relatively long elimination half-life. A possible prolonged biological effect of this drug may explain why all infections were of low intensity. It is difficult to ascertain the variable effect of antimalarials on efficacy over time since the study was cut short by rains. Finally, naturally-acquired host immunity may have helped to clear merozoites emerging from the liver, possibly creating a synergistic effect between vaccine-induced and naturally-acquired responses.

### 4.4 Combination vaccine approaches

The current leading malaria candidate, RTS,S/AS01, has completed Phase III efficacy trials in the target population of children and infants in sub-Saharan Africa [79]. Efficacy data are now available on a median follow-up time of 36 months for young infants receiving their first dose of RTS,S/AS01 at 6–12 weeks of age (“infants”), and for the older infants and children who received their first vaccination at 5–17 months of age (“children”). Efficacy against clinical malaria in infants was 18% [12–24% CI] and against severe malaria clinical malaria, a non-significant 10% [−18% to 32%]. So, in this group that received other Expanded Programme on Immunization (EPI) vaccinations, overall efficacy was modest. This could be improved to 26% (clinical) and 17% (severe malaria) by administering a booster vaccination dose at month 20. In the older age group of children, efficacy against clinical malaria over 48 months median follow-up was 28% increasing to 36% will a booster dose; however, efficacy against severe malaria was only 1% without a booster, and 32% with the booster dose.

These modest levels of protection over 3–4 years conceal much higher levels of efficacy in the first months after immunization that are associated with very high levels of antibody to the central repeat of the CS protein. These levels of hundreds of ELISA Units per mL fall over time and protection wanes. One approach to attempt to improve durability and overall efficacy could be to add a viral vector encoding ME-TRAP to RTS,S/AS01 in a combination approach, and Phase I/IIa trials of this approach are in progress as a collaboration between Oxford and GSK Vaccines. Moreover, once efficacy is achieved with other stage components, *e.g. Pf*RH5, one strategy will be to attempt to combine several partially protective vaccine components into a higher efficacy multi-stage vaccine.

### 4.5 “Unnatural immunity”

It is striking that the levels of antibody response to CS protein induced by RTS,S/AS01 are about 100 fold greater after immunization than is ever observed pre-vaccination or after natural malaria exposure. Similarly, the levels of T cell response induced by viral vectors to TRAP are about 100-fold greater than those found after a lifetime of natural exposure to malaria. But these very high levels of immune response are required for partial protection against malaria. Interestingly, immune responses to the promising blood-stage vaccine candidate antigen *Pf*RH5 are also modest after natural exposure so that, again, the current *Pf*RH5 vaccine candidates should induce “unnatural” levels of immunity. Finally, natural immune responses to the leading transmission-blocking candidate antigen, *Pf*s25, are minimal (or non-existent due to lack of antigen expression in the mammalian host) so that again vaccination induces immune responses that are never observed naturally. Clearly, modern vaccine technology allows immune responses that are “better than nature” to be induced, but an on-going challenge will be to identify these key responses and maintain protective levels to provide durability of efficacy.

In addition to demonstrating the impressive immunogenicity and level of efficacy attainable with viral vectors, work is on-going to further demonstrate safety of these relatively new vectors in diverse populations. These include children and infants in African countries including young infants receiving simultaneously other EPI vaccines. To date over a thousand subjects have been immunized with ChAd vectors for malaria with a very good safety record and even larger numbers have been immunized with MVA vectors encoding malaria antigens. This goal of safety assessment is supported by the increasingly widespread use of other simian adenovirus and MVA vectors for vaccination against tuberculosis [80], HIV [81], Ebola [82] and other diseases.

*Conflict of interest*: None of the authors has conflicts of interest.

## Figures and Tables

**Table 1:** Summary of clinical trials employing viral vectors for malaria since 2010.



